# The role of resilience in daily experiences of posttraumatic growth, affect, and HIV/AIDS stigma among people living with HIV

**DOI:** 10.1038/s41598-023-28187-x

**Published:** 2023-01-16

**Authors:** Małgorzata Pięta, Marcin Rzeszutek

**Affiliations:** Faculty of Psychology, University of Warsaw, Stawki 5/7, 00-183, Warsaw, Poland

**Keywords:** Quality of life, Psychology, HIV infections

## Abstract

This study investigated the intraindividual variability in daily posttraumatic growth (PTG) versus posttraumatic depreciation (PTD), positive and negative affect (PA and NA), and HIV/AIDS stigma among people living with HIV (PLWH). In particular, we examined whether this variability derives from participants’ resilience operationalized on a trait level. The participants included 67 PLWH, who completed a baseline questionnaire on resilience, measured with the Brief Resilience Scale. Following this, they completed the shortened versions of the following inventories over five consecutive days: the Posttraumatic Growth and Posttraumatic Depreciation Inventory V Expanded version Inventory, the Positive and Negative Affect Schedule – Expanded Form, and the Berger HIV Stigma Scale. Hierarchical linear modeling (HLM) was utilized to analyze the study results. We observed significant intraindividual variability in PTG, PTD, PA, NA, and perceived HIV/AIDS stigma. Resilience was related to PTG, PTD, PA, and NA but not to stigma. Moreover, higher resilience was associated with higher, stabler PA and lower, stabler NA. Our results highlight the need of further studies on the daily functioning of PLWH. Specifically, while health status is important, it does not appear to be the predominant source of everyday distress for PLWH. Consequently, psychological counselling for PLWH should concentrate more on the life of the person as a whole and not only on coping with HIV infection.

## Introduction

Stories of growth after trauma and adversity are common throughout human history and across various cultures and historical epochs^1^. Similarly, despite the relatively recent emergence of posttraumatic growth (PTG) as a research area^2,3^, studies on PTG have proliferated quickly, creating a vast body of evidence supporting the meaningful, positive changes following traumatic or adverse life events that have been suggested historically^1^. Nevertheless, while the interest in PTG has contributed to significant development of this field, several questions remain unanswered concerning the objective manifestations and stability of PTG as well as the mechanisms underlying positive changes^4^. Specifically, various limitations of classic PTG study designs and their assessment have prevented a thorough explanation of these phenomena^5^. The most commonly used PTG measures, such as the Posttraumatic Growth Inventory (PTGI)^2^, tend to evaluate PTG almost exclusively based on self-reports and retrospection^6^. Additionally, PTG evaluation based on participants’ subjective recollections is a cognitively demanding procedure prone to significant biases, especially when studied only cross-sectionally^7^. Nevertheless, the extensive critique of previous PTG research has inspired a search for methodological advancements in this field and multimethod approaches for studying and measuring PTG^5^.

First, the inclusion of parallel items that reflect negative changes in standard PTG tools has been suggested as a way to overcome positivity bias in PTG measures that focus exclusively on growth^8,9^. This led to the emergence of a new construct of posttraumatic depreciation (PTD), revealing some counteractive patterns of posttraumatic change, such as the simultaneous occurrence of PTG and PTD, as well as different predictors shared among various populations after trauma^10,11^. Second, the use of longitudinal study designs has been proposed to answer questions regarding potential PTG dynamics over time^4^. Finally, to overcome the limitations of retrospective PTG reports and related biases, authors have increasingly advised more ecological, daily PTG assessments^6^. In particular, intensive longitudinal measurement using experience sampling methods or electronic daily diaries has been suggested as a way to verify whether PTG manifests in people’s everyday lives after trauma or is only a retrospective, illusory belief among trauma survivors^12–14^. In other words, a new type of PTG measurement could elucidate short- and long-term PTG dynamics, offering key insights for implementing PTG-focused clinical interventions^6,15^.

Moreover, the noted limitations in this field have also led to several ambiguities concerning how to identify PTG predictors^16^. One such ambiguity concerns the relationship between PTG and resilience. We do not know if the constructs’ overlap or whether PTG is supported or inhibited by the baseline resilience levels of individuals who have experienced traumatic life events^17,18^. Although resilience can be considered a PTG-promoting factor, it may also reinforce resistance to negative emotions and promote “bouncing back” to baseline levels of functioning after trauma rather than growth^13,19^. These processes have been particularly understudied in the context of the daily manifestations of resilience and its impact on psychological well-being during stress and adversity^20,21^. Psychological resilience can be understood as a stable trait or an ability that supports both the maintenance and generation of positive emotions during stressful situations^19,22^. Still, since it occurs within the PTG context, few studies have used intensive measurement methods to track daily manifestations of resilient functioning during stress or trauma (e.g.,^6,23–26^). Similarly, no previous research has examined the daily within-person variability of PTG and its association with baseline resilience levels in trauma-affected populations. Parallel measurement of PTG and resilience may provide a better understanding of this association and its clinical implications for specific populations following health-related trauma, including people living with HIV (PLWH)^27^.

Investigating PTG among PLWH may shed new light on the process of adaptation to HIV infection^28^. Several studies have demonstrated the link between PTG, better mental health, and increased adherence to treatment among PLWH (see metanalysis^27^). However, to better understand this process, it is necessary to explore the mutual association between PTG and resilience in this clinical sample^29–31^. In particular, the extent to which these constructs overlap with each other or the degree to which they influence one another in this sample are unknown^30,31^. Consequently, some authors have suggested the need to analyze environmental variables, such as HIV/AIDS stigma^29,32^, especially based on daily assessments^33^.

The daily life experiences of PLWH remain a relatively understudied research area^34–36^. However, for individuals experiencing chronic but manageable and non-acute stress conditions, such as living with HIV, monitoring of their affective states is crucial^34^. Vulnerable clinical populations are subjected to dynamic stress circumstances, such as HIV/AIDS stigma, which should be monitored in these patients’ daily lives^33^. A particularly interesting question is whether daily fluctuations in PLWH’s positive and negative well-being are associated with their baseline levels of various stable personal characteristics^33,34^. Accordingly, we focused on assessing the role of psychological resilience in the daily lives of PLWH in this study.

Specifically, this study aimed to investigate intraindividual variability in daily PTG and PTD levels, self-reported affect (PA/NA), and HIV/AIDS stigma intensity among PLWH. Additionally, we sought to examine whether this variability on a state level derives from resilience on a trait level, which was measured on the first day of the study. To the best of our knowledge, no previous studies have examined such factors among PLWH using this particular study design and these specific variables, from which we derived our hypotheses. Thus, our study is mainly explorative. However, based on research on the daily psychosocial functioning of PLWH (e.g.^34–37^), the association between resilience measurements and intraindividual fluctuations in affective well-being in the general population^20,21^, and research on HIV/AIDS stigma^38^, we formulated the following hypotheses:

*Hypothesis 1*. PLWH experience intra-individual variability in their daily reported levels of PTG and PTD, PA and NA, and perceived HIV/AIDS stigma.

*Hypothesis 2*. The intra-individual variability in PLWH’s daily reported levels of PTG and PTD, PA and NA, and perceived HIV/AIDS stigma measured at the state level is related to resilience at the trait level.

## Methods

### Participants and procedure

Sixty-seven patients who had been diagnosed with HIV were included in this study. The measurements were conducted at the beginning of 2021 as part of a larger project, for which the data collection was finalized at the beginning of 2022. Of the patients who provided their contact information on the paper-and-pencil survey, 67 who did not drop out from any measurements were chosen for further participation and analysis in this study, from whom 14 (20.9%) were women. The mean age of the participants was 40.76 (SD = 11.28). 32 (47.8%) participants stayed in a stable relationship. The minority, i.e., 15 (22.4%) participants, was heterosexual and the rest of the sample was of homosexual orientation. 13 (19.4%) PWLH had an ongoing problem with substance misuse. 40 (59.7%) participants received higher education, 25 (37.3%) identified with secondary education, while 2 (3%) persons had primary or vocational schooling. More than a half of participants (43; 64.2%) had regular employment, while the rest was unemployed (11; 16.4%), received a pension (10; 14.9%) or retired (3; 4.5%). 11 (16.4%) participants described their financial situation as very good, 28 (41.8%) stated it was good, 11 (29.9%)—medium. 6 (9%) participants described their situation as bad, and 2 (3%) as very bad. 9 (13.4%) PLWH entered the AIDS stage. 5 (7.5%) participants had a detectable viral load. The mean ARV treatment time among the study sample was 7.07 years (SD = 5.89). Table 1 presents the demographic characteristics of the study sample.

**Table 1:** Participants’ demographic characteristics (n = 67).

		*n*	%
Gender	Women	14	20.9
	Men	53	79.1
Age		23–73	M = 40.76; SD = 11.28
Relationship	In a stable relationship	32	47.8
Education	Primary	1	1.5
	Vocational	1	1.5
	Secondary	25	37.3
	Higher	40	59.7
Employment	Regular employment	43	64.2
	Unemployed	11	16.4
	Pension	10	14.9
	Retired	3	4.5
Subjective financial status	Very good	11	16.4
	Good	28	41.8
	Medium	20	29.9
	Bad	6	9.0
	Very bad	2	3.0
Sexual orientation	Heterosexual	15	22.4
	Homosexual	46	68.7
	Other	6	9.0
Addiction	Ongoing use	13	19.4
AIDS	Diagnosed	9	13.4
Viral load	Detectable	5	7.5
ARV treatment	In years	0.50–30	M = 7.07; SD = 5.89

*M* mean value, *SD* standard deviation, *n* number of participants in a category.

Participants were recruited from Warsaw’s largest healthcare clinic for PLWH. During the initial measurement session, participants completed a paper-and-pencil version of the psychometric questionnaires (see Measures), including the sociomedical survey. They were also asked to provide their email address or telephone number for further contact during the diary part of the study. Only participants who agreed to provide their contact information were included in this further part of the study. Participants were informed that involvement in the study was voluntary and that no remuneration would be provided. Medical doctors also assessed patients for further eligibility criteria, such as being at least 18 years old, having a medical HIV infection diagnosis, having undergone antiretroviral treatment, and having no cognitive disorders. The viral suppression and entering the AIDS phase of HIV infection, were controlled throughout a one-year period as part of a larger longitudinal project. These two health parameters remained stable among study participants that received complex healthcare in specialized HIV clinic in Warsaw, Poland. The whole project consisted of three classical and three diary measurements that took place in aftermath of the first stage described in the article.

In the preliminary stage of this study, patients’ characteristics, such as their socio-demographic and medical data, and resilience levels were measured at a trait level (see the following two subsections). For the electronic daily diary measurements, we prepared shortened electronic versions of the questionnaires measuring state-like variables (PTG and PTD, PA and NA, and HIV/AIDS stigma) using a special online platform. Participants were asked to complete electronic diaries for five consecutive days (Monday to Friday). The diaries were provided by e-mail at the end of each study day. Links were sent to participants at 6:00 PM, followed by a reminder at 9:00 PM. On each study day, these links stayed active until 1:00 AM the following day, at which point they were deactivated. Participants were invited to contact the study organizers via email or telephone throughout the whole study period if they needed any technical help accessing the diaries.

The study methodology was approved by the ethical committee of the Faculty of Psychology of the University of Warsaw, and the study was carried out in accordance to relevant guidelines and regulations. Informed verbal consent was obtained from all participants. Informed verbal consent was obtained from all participants and this procedure was approved by the ethics committee of University of Warsaw.

## Materials

### Trait-level measurement

#### The Brief Resilience Scale (BRS)

The Polish adaptation of the Brief Resilience Scale (BRS) scale^19^ was used in this study as the paper-and-pencil measurement of the participants’ trait-level resilience. BRS is a six-item scale that employs a five-point Likert response, ranging from 1 (*strongly disagree*) to 5 (*strongly agree*). The Cronbach’s alpha for the total resilience score was satisfactory (see Table 2).

**Table 2:** Descriptive statistics of analyzed variables among participants.

Day	Variables	M	SD	min	max	S	K	α
Day 1	Resilience	21.35	5.97	9.00	30.00	− 0.45	− 0.76	0.92
	Positive affect	2.78	0.89	1.00	4.67	0.18	− 0.20	0.88
	Negative affect	2.08	0.93	1.00	5.00	0.11	0.67	0.91
	PTG	2.98	1.07	0.00	5.00	− 0.70	0.53	0.76
	PTD	1.21	1.16	0.00	4.20	0.10	0.18	0.82
	Stigma	1.78	0.77	1.00	4.00	0.16	0.67	0.91
Day 2	Positive affect	2.86	0.90	1.00	4.67	− 0.23	− 0.38	0.86
	Negative affect	1.94	0.90	1.00	5.00	0.59	0.53	0.89
	PTG	2.97	1.06	0.00	4.80	− 0.84	0.80	0.75
	PTD	1.03	1.05	0.00	4.00	0.21	0.89	0.79
	Stigma	1.74	0.77	1.00	4.00	0.09	0.76	0.92
Day 3	Positive affect	2.82	0.82	1.00	4.67	0.08	− 0.23	0.83
	Negative affect	1.97	0.84	1.00	4.83	0.52	0.13	0.87
	PTG	2.99	1.02	0.00	5.00	− 0.33	0.01	0.73
	PTD	1.02	0.94	0.00	4.00	0.94	0.63	0.70
	Stigma	1.67	0.72	1.00	4.00	0.18	0.24	0.89
Day 4	Positive affect	2.97	0.80	1.00	4.83	− 0.63	0.57	0.83
	Negative affect	2.02	1.05	1.00	5.00	0.24	0.61	0.93
	PTG	3.03	1.00	0.80	5.00	− 0.42	− 0.39	0.71
	PTD	1.17	1.15	0.00	4.00	0.01	− 0.02	0.82
	Stigma	1.70	0.78	1.00	4.00	0.15	0.92	0.92
Day 5	Positive affect	2.94	0.90	1.00	4.50	− 0.07	− 0.67	0.87
	Negative affect	2.02	0.97	1.00	5.00	0.26	0.18	0.92
	PTG	2.99	1.02	0.00	4.40	− 0.94	0.06	0.74
	PTD	1.17	1.08	0.00	5.00	0.39	0.24	0.80
	Stigma	1.66	0.76	1.00	4.00	0.14	0.87	0.92

*M* mean value, *SD* standard deviation, *min* minimum value, *max* maximum value, *S* skewness, *K* kurtosis; *α* Cronbach’s α reliability coefficient.

### State-level measurement

#### Shortened version of the posttraumatic growth and posttraumatic depreciation inventory: expanded version (PTGDI-X)

PTG and PTD were measured with a 10-item scale, five of which measured each construct in the 50-item PTG and PTD Inventory (PTGDI-X)^39^. We chose 10 items, one from each of five subscales of PTG and PTD measures, which most strongly loaded on the relevant global PTG and PTD factors. Responses ranged from 0 (*I did not experience this change*) to 5 (*I experienced this change to a great degree*). Higher scores indicate more intense PTG or PTD levels. We did not count the subscale indicators. Instead, we calculated the global PTG and PTD scores, which reflect the sum of all items in the PTG and PTD domains, respectively. Participants were instructed to focus on daily positive and negative experiences in their lives after receiving their HIV diagnosis, such as “I felt that I have numerous opportunities” or “I felt that I cannot change much in my life.” We used this state-level measure of PTG and PTD following the recommendation by Blackie et al.^6^. The Cronbach’s alphas for the global PTG and PTD scores were satisfactory (see Table 2).

#### The shortened version of the positive and negative affect schedule-expanded form (PANAS-X)

Participants reported the affective states they experienced during a study day using a five-point Likert scale ranging from 1 (*not at all*) to 5 (*strongly*). A list of 12 feelings and emotions was provided. We chose an equal number of items from the PA and NA subscales, with six for PA (e.g., “satisfied,” “energetic”) and six for NA (e.g., “angry,” “worried”), which were also strongly loaded on the PA and NA scales^40^. The Cronbach’s alpha coefficients obtained in this study were satisfactory (see Table 2).

#### The Berger HIV stigma scale (HSS)

The shortened version of the Polish adaptation of the HIV/AIDS stigma inventory (Berger HIV Stigma Scale [HSS])^41^ was used to measure daily experiences of stigma. As for the previous measures, the most representative, strongly loaded items from with subscale were selected. This electronic inventory consisted of five items, such as “I felt that others who know or may know about my HIV infection may have had a bad opinion about me.” Answers were provided using a Likert scale ranging from 1 (*strongly disagree*) to 4 (*strongly agree*). In this study, the total HIV/AIDS score was used. The Cronbach’s alpha for the total stigma score was satisfactory (see Table 2).

### Data analysis

During our preliminary analysis, we calculated the descriptive statistics. Next, hierarchical linear modeling (HLM) was used to assess the participants’ intra-individual variability in PTG and PTD, PA and NA, and stigma levels over five consecutive days as well as its relationship to interindividual differences in resilience, which were measured using the scales described in the previous subsections. HLM (known as *multilevel modeling*) is a statistical method that uses ordinary least square (OLS) regression-based analysis to measure the hierarchical structure of data^42^. Hierarchically structured results are nested data, where groups of units are clustered together in a specific pattern, which vary at more than one level.

PTG and PTD, PA and NA, and levels of HIV/AIDS stigma over five consecutive days were analyzed as dependent variables. Each dependent variable was analyzed in a separate model. The measurements—for example, time variables in days—were analyzed as covariates and fixed effects. Random effects for intercepts and temporal effects were also included. Each model was analyzed twice: with and without resilience levels included as a covariate. This solution allowed for comparisons regarding the variance in results acquired across consecutive days when resilience was and was not considered. The models that included resilience also included also the fixed effects of resilience and resilience over time.

### Ethical approal

All procedures performed in studies involving human participants were in accordance with the ethical standards of the institutional and/or national research committee and with the 1964 Helsinki Declaration and its later amendments or comparable ethical standards.

## Results

Table 2 presents descriptive statistics for the analyzed variables. All variables were measured with adequate reliability. The values of skewness and kurtosis did not exceed a range of − 1.0 to 1.0; therefore, HLM based on ordinary least square method could be applied. No substantial differences between mean values acquired in the consecutive measurements were observed.

The main analysis was performed using the HLM. The results of this analysis are depicted in Table 3.

**Table 3:** Relationships between resilience, time, and daily levels of PTG or PTD, positive affect, negative affect, and HIV/AIDS stigma among participants.

Dependent variable	Resilience included			
			Estimate	*p*
PTG	No	Time	− 0.006	0.840
		Time variance	0.017	0.063
	Yes	Time	0.007	0.949
		Resilience	0.065	0.003
		Resilience × time	− 0.001	0.883
		Time variance	0.018	0.052
PTD	No	Time	0.033	0.931
		Time variance	0.020	0.040
	Yes	Time	− 0.041	0.734
		Resilience	− 0.059	0.009
		Resilience × time	0.002	0.710
		Time variance	0.021	0.032
Positive affect	No	Time	0.043	0.124
		Time variance	0.003	0.616
	Yes	Time	0.158	0.124
		Resilience	0.068	0.001
		Resilience × time	− 0.006	0.220
		Time variance	0.002	0.650
Negative affect	No	Time	− 0.006	0.839
		Time variance	0.015	0.068
	Yes	Time	− 0.102	0.364
		Resilience	− 0.051	0.008
		Resilience × time	0.005	0.358
		Time variance	0.011	0.093
Stigma	No	Time	− 0.027	0.082
		Time variance	0.006	0.045
	Yes	Time	− 0.023	0.690
		Resilience	− 0.015	0.339
		Resilience × time	0.000	0.910
		Time variance	0.006	0.045

**Figure 1:**
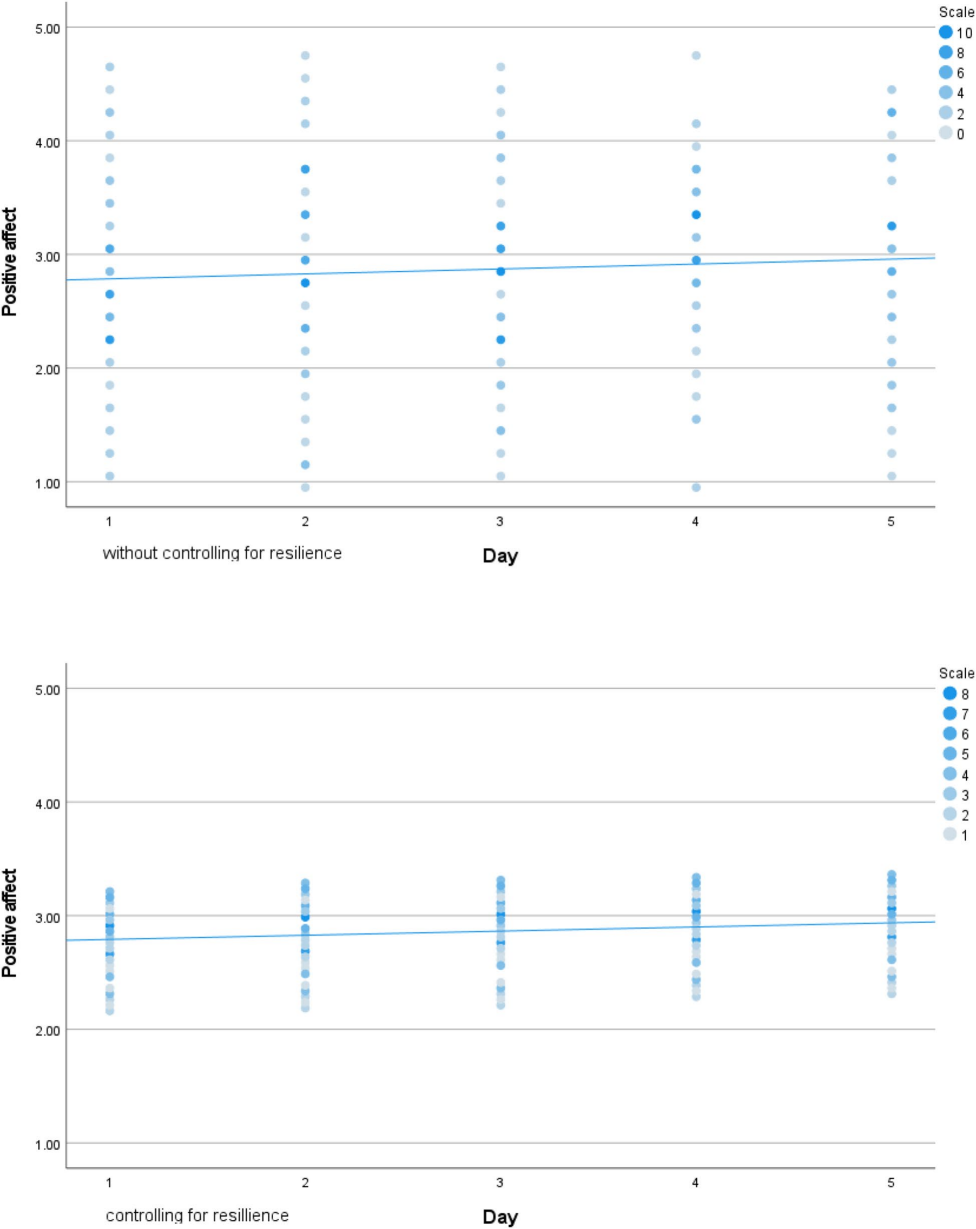
Variance in positive affect in five consecutive days without and with controlling for baseline resilience level.

**Figure 2:**
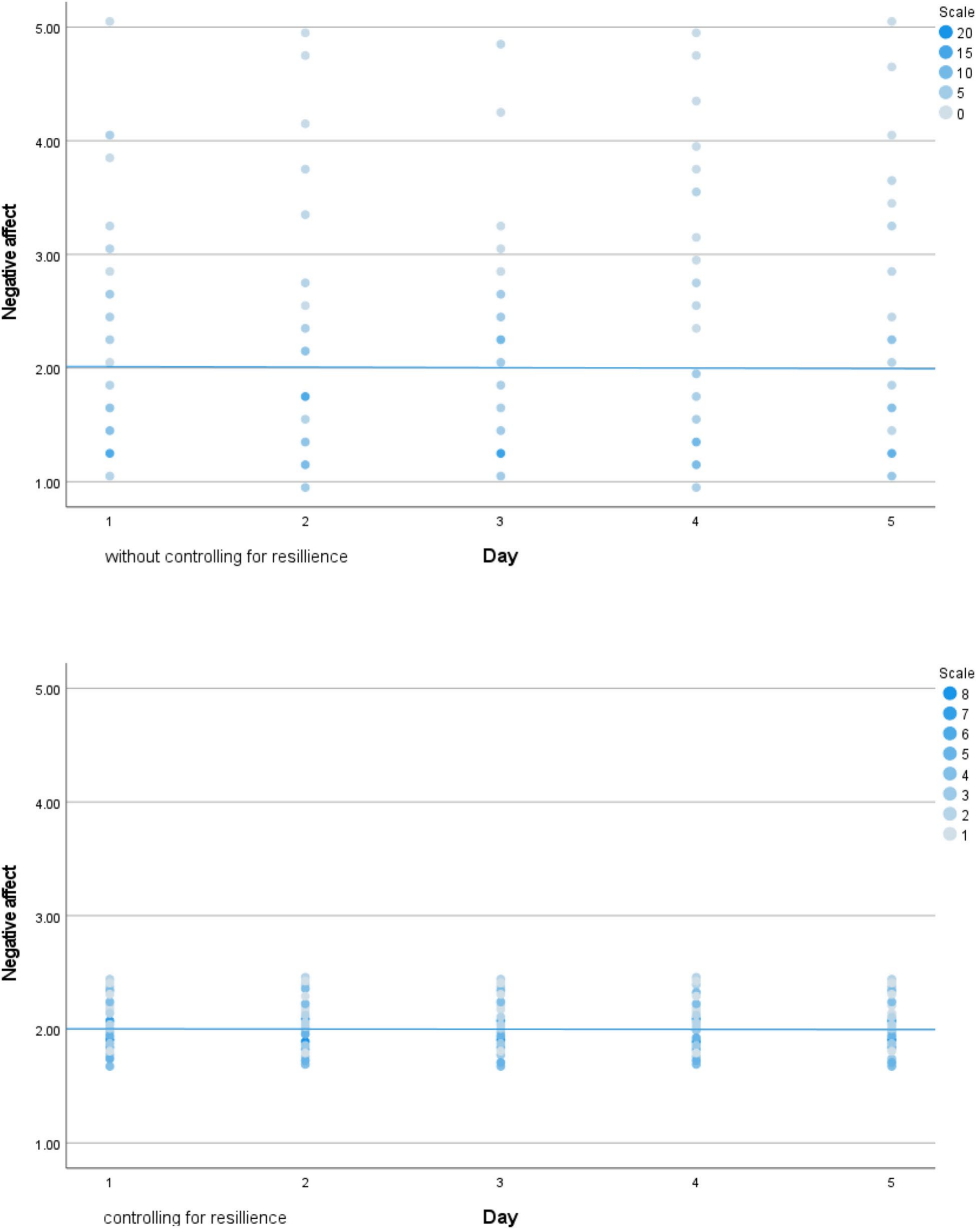
Variance in negative affect in five consecutive days without and with controlling for baseline resilience level.

No statistical effect of time was observed which means that there were no statistical differences between the consecutive days in general. Statistically significant positive relationships were found between resilience levels, average PTG levels, and PA over five consecutive days. Also, significant negative relationships were found between resilience levels, average NA levels, and PTD over five consecutive days. No statistically significant relationship was found between resilience levels and average stigma levels. The values of estimated variance fell after resilience was included in calculating PA and NA. A corresponding effect was not observed in our analysis of PTG, PTD, and stigma. Additionally, resilience levels were important in determining the dynamics of affect changes, such that higher resilience was associated with higher, stabler PA and lower, stabler NA. In contrast, resilience levels were related to PTG and PTD’s daily intensity (see above), but they did not affect the stabilization of day-to-day changes. The values of estimates of variance dropped after adding resilience in case of positive affect and negative affect are presented at Figs. 1 and 2).

## Discussion

The results of our study mostly supported our first hypothesis, as we observed significant intraindividual variability in daily PTG and PTD, PA and NA, and perceived HIV/AIDS stigma levels among participants. Our findings contribute to the literature on PLWH’s daily functioning, an understudied subject, as our analysis was not limited to coping with HIV infection but rather examined the everyday lives of PLWH^35,43–45^. Previous studies on this topic have focused on rather narrow aspects of PLWH’s function, predominantly HIV symptoms, substance use and abuse, or adherence to treatment^38^. In other words, those studies were conducted under the implicit assumption that PLWH are entirely preoccupied with their HIV-positive status and its associated distress^46^. However, great progress in treatment has changed HIV infection from a fatal to a chronic, manageable health condition^47^. Thus, health status remains important but is not necessarily the main cause of PLWH’s stress and well-being issues^35^. Despite sharing a source of distress—their HIV-positive status—PLWH’s psychological functioning may fluctuate daily, and these fluctuations may be associated with significant individual differences in their psychological functioning over time^36,48^. Studies employing an intensive longitudinal study design, assessing behaviors as they occur in real time in an individual’s natural surroundings, may increase ecological validity and provide a unique opportunity to monitor individual differences among this patient group^12,35^.

Within the PTG literature, our study is the first to identify daily manifestations of this positive phenomenon in the clinical population. Previously, only Blackie et al.^6^ showed significant within-person variability in daily state-level PTG among a sample of college students after adverse life events. Measuring PTG daily could offer important insights on the longstanding debate regarding PTG’s real versus illusory nature, which has been predominantly assessed via retrospective questionnaires^4,7,14^. It remains unknown whether any stable changes (positive or negative) really occur after trauma or whether these changes can occur in daily life and be identified retrospectively through self-reports. More studies assessing PTG daily are needed to prove whether PTG is just a trait-like tendency to retrospectively declare positive changes following adversity or whether it can be operationalized as a state-like term that manifests in trauma survivors’ day-to-day behaviors^6,13^.

The results of the current study also supported our second hypothesis to an extent. On the one hand, we found respectively positive and negative relationships between resilience levels and average PTG and PTD levels among participants. A similar association was observed for affective well-being: a positive relationship with PA and a negative relationship with NA. However, we noted that resilience levels were important for affect alone, and higher resilience was linked to higher, stabler PA and lower, stabler NA. Concerning PTG and PTD, we found no such stabilization of day-to-day changes as a result of resilience. Further, we observed no relationship between daily HIV/AIDS stigma intensity and resilience. Our findings contribute to the long-term debate on the status of psychological resilience (e.g.^18,19,49,50^). In particular, whether resilience should be operationalized and measured as a static trait or as a dynamic state remains unknown, as do the consequences of these operationalizations for the association between resilience and well-being across various samples following stressful and adverse life events^20^. These ambiguous findings are partially linked to the measurement limitations of resilience studies, which have mainly employed cross-sectional designs and focused only on significant life challenges. Almost no research has been conducted on the association between resilience and daily stressors and well-being outcomes^20^. Previously, only Ong et al.^21^ found that psychological resilience assessed on the trait level is related to intraindividual variability in daily emotional responses to stress among older adults, with higher trait resilience predicting faster recovery from daily stress due to a high level of positive emotions. Moreover, Ong et al.^21^ claimed that, although positive emotions are a fundamental feature of trait resilience, they cannot be reduced to a simple byproduct of resilience^50^. They found that resilient individuals often adopt positive emotion-eliciting coping strategies, such as benefit finding and positive reappraisal, which regulate their negative affective experiences^50^. Overall, high resilience seems to give individuals greater access to momentary positive emotional resources, which are also stabler over time, thus protecting them better from daily stress compared to less resilient people^21^. Additionally, resilience may influence levels of emotional complexity, promoting greater differentiation, control, and separation between PA and NA when experiencing stress in daily life^51,52^.

Finally, our study is the first to observe the relationship between resilience assessed on the trait level and PTG operationalized daily. Thus, it offers an important contribution to the ongoing debate on the association between resilience and PTG, particularly among PLWH, an issue that has elicited mixed views (see:^29–31^). Moreover, the lack of a link between trait-level resilience and daily stigma may suggest that HIV/AIDS stigma is a dynamic and transient phenomenon that is not rooted in the individual, stable characteristics of PLWH but rather associated with external social conditions^33^. In other words, regardless of intrinsic factors, for PLWH stigma is highly situational or environmental. This finding may suggest possible interventions, such as focusing less on addressing a person’s individual traits and more on providing positive social support and improving the patient’s external environment as well as removing the social stigma and harmful prejudice associated with living with HIV.

### Strengths and limitations

This study had several strengths, including its two modes of variable measurement (trait- and state-level measurement), intensive longitudinal design, and clinical sample of PLWH. However, a few limitations should be noted. First, although we employed an intensive, longitudinal design, this study was correlational, so we cannot draw any cause-and-effect explanations. Second, this study’s highly explanatory character complicates any discussion of its implications for the wider context of PLWH’s lives. Third, we controlled for a relatively small amount of sociodemographic and HIV-related clinical variables, and our sample was heterogenous with regard to HIV infection duration. Further, we restricted our research to a sample of PLWH who were receiving complex medical care and following ART treatment. Consequently, our participants should be viewed as highly functional PLWH. Future studies should focus on more heterogeneous samples of PLWH with respect to their socioeconomic characteristics and HIV infection progression.

### Calls for further research

The results of our study suggest the need for further advancements in PTG operationalization and measurement, which should focus on identifying the daily manifestations of PTG in real life and its intraindividual variability^5,6^. Such research could determine whether PTG is simply a trait-like tendency to retrospectively declare positive changes following adversity or if it can be operationalized as a state-like phenomenon that manifests in trauma survivors’ day-to-day behaviors. In this study, we already outlined some potential associations between resilience and daily PTG or PTD fluctuations. However, further scientific inquiry is necessary to differentiate between the stable and situational factors that may promote or hinder stable positive changes amid adversity.

From the perspective of PLWH, our results call for further studies on the still-neglected subject of PLWH’s daily functioning that are not only focused on coping with HIV infection but also consider various areas of functioning^35^. In particular, affective functioning may prove to be indicative of more stable individual differences in psychological functioning associated with some of the major mental health challenges PLWH face^36^. Consequently, researchers should employ intensive longitudinal designs to better identify dynamic individual differences in PLWH’s well-being.

## Supplementary Information

Supplementary Information 1.

## Data Availability

All data generated or analyzed during this study are included in this published article (and its supplementary information files).
