# Author Correction: The role of resilience in daily experiences of posttraumatic growth, affect, and HIV/AIDS stigma among people living with HIV

**DOI:** 10.1038/s41598-023-36598-z

**Published:** 2023-06-09

**Authors:** Małgorzata Pięta, Marcin Rzeszutek

**Affiliations:** Faculty of Psychology, University of Warsaw, Stawki 5/7, 00‑183 Warsaw, Poland

Correction to: *Scientific Reports* 10.1038/s41598-023-28187-x, published online 16 January 2023

The original version of this Article contained an error, where the incorrect file was provided as the Supplementary Information 1 file.

The Supplementary Information 1 file that accompanies the original Article has now been corrected.

